# Evidence-Based Nutraceuticals Derived from *Antrodia cinnamomea*

**DOI:** 10.3390/foods14071212

**Published:** 2025-03-30

**Authors:** Chunyuhang Xu, Qingtong Xie, Chien-Liang Kuo, Xin Yang, Dejian Huang

**Affiliations:** 1 Department of Food Science and Technology, National University of Singapore, 2 Science Drive 2, Singapore 117542, Singapore; e0669581@u.nus.edu (C.X.); e0966402@u.nus.edu (Q.X.); 2 PhD Programme for Aging, College of Medicine, China Medical University, Taichung 406040, China; cl.kcll@gmail.com; 3 David H. Koch Institute for Integrative Cancer Research, Massachusetts Institute of Technology, Cambridge, MA 02139, USA; 4 National University of Singapore (Suzhou) Research Institute, 377 Linquan Street, Suzhou 215123, China

**Keywords:** *Antrodia cinnamomea*, medicinal mushroom, nutraceuticals, functional foods, terpenoids

## Abstract

*Antrodia cinnamomea* (*A. cinnamomea*), a medicinal and edible mushroom endemic to Taiwan, has been traditionally valued as a health tonic. Recent studies have highlighted the diverse specialized metabolites and bioactive potential of this substance, primarily attributed to key secondary metabolites such as benzenoids, maleic and succinic acids, ubiquinone, triterpenoids, and the primary metabolite polysaccharides. These compounds exhibit a broad spectrum of pharmacological properties, including those related to antibacterial, antitumor, anti-inflammation, hepatoprotection, hypoglycaemia, and antioxidant activities, and immunomodulation and gut microbiota regulation. These findings highlight the therapeutic potential of *A. cinnamomea* and its potential applications in health supplements and functional foods. This review evaluated recent advancements in the cultivation, extraction, and characterization of bioactive compounds from *A. cinnamomea*, with a particular focus on submerged and solid-state fermentation methods. We hope to provide a comprehensive framework for promoting the efficient and scientific evidence based utilization of *A. cinnamomea* in novel therapeutic strategies and health-related innovations.

## 1. Introduction

*Antrodia cinnamomea* (commonly known as *“Niu-Chang-Chih”* in Chinese) is a highly esteemed edible and medicinal mushroom native to Taiwan traditionally used as a health tonic by aboriginal tribes and the broader Asian community [1,2]. Wild *A. cinnamomea* has been used medicinally in Taiwan for a long time, particularly by aboriginal tribes [3]. *A. cinnamomea* was used to treat abdominal pain, food poisoning, diarrhoea, and itchy skin and to enhance liver function [4]. Recent research efforts have led to the isolation and identification of numerous chemical constituents from *A. cinnamomea*, and an increasing numbers of human studies have been reported on anticancer, antioxidant, antihypertensive, hypolipidemic, immunomodulatory, and anti-inflammatory functions, which were believed to be due to benzenoids, maleic and succinic acids, ubiquinone, triterpenoids, and polysaccharides [5,6]. The host of the mushroom, the decomposing trunks of *Cinnamomum kanehirai*, is an endangered tree species found in Taiwan’s broad-leaved forests at altitudes ranging from 450 to 2000 m above sea level [7]. This limited natural habitat, combined with the slow growth rate of *A. cinnamomea*, has earned it the moniker “ruby of the forest” [3]. Due to its value as a high-quality raw material for furniture and the therapeutic potential of *A. cinnamomea* cultivated on it, *Cinnamomum kanehirai* has been subject to extensive logging, and this exploitation has prompted Taiwan to place *C. kanehirai* on its endangered species list [3]. With the increasing demand, wild *A. cinnamomea* can no longer meet market needs, driving attention toward artificial cultivation methods of artificial cultivation include basswood cultivation, solid-state cultivation, and submerged fermentation, which closely replicates wild substrate quality [8]. With advancements in research, investigations into *A. cinnamomea* have progressed beyond crude extracts, focusing on isolated bioactive compounds such as polysaccharides, triterpenoids, and ubiquinone derivatives from its fruiting bodies and cultured mycelia.

## 2. Cultivation and Extraction Methods for *A cinnamomea*

### 2.1. Cultivation Methods

**Figure 1:**
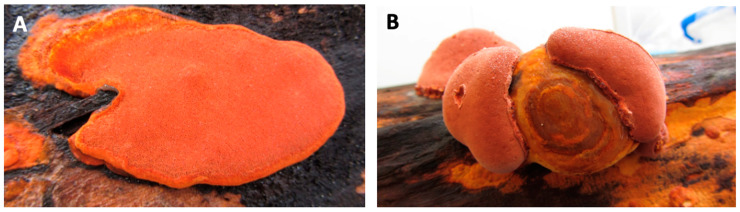
Pictures of *Antrodia cinnamomea*. (**A**) Typical flat type wild fruiting bodies; (**B**) Horse-hoof-shaped wild fruiting bodies. Pictures of *Antrodia cinnamomea*. (**A**) Typical flat type wild fruiting bodies; (**B**) Horse-hoof-shaped wild fruiting bodies. (Images provided by Dr Chien-Liang Kuo from *AgriGADA*).

**Figure 2:**
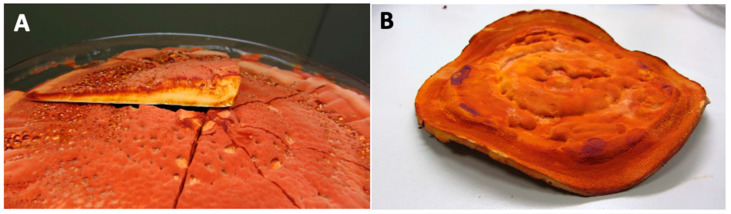
Pictures of *Antrodia cinnamomea* dish cultures. (**A**) Cross section of Antrodia cinnamomea; (**B**) whole photo of dish cultures. (Images provided by Dr Chien-Liang Kuo from *AgriGADA*).

Artificial cultivation methods of *A. cinnamomea* include basswood cultivation, plate culture, solid-state fermentation, and submerged fermentation (Figure 1 and Figure 2).

#### 2.1.1. Liquid Fermentation

Liquid fermentation is a straightforward and efficient method for cultivating *A. cinnamomea* mycelium. It utilizes a nutrient-rich liquid medium often enhanced with natural botanical extracts, which is cost-effective, easy to implement, and can enhance secondary metabolite synthesis [9]. The liquid fermentation method offers significantly shorter cultivation periods, typically lasting only a few weeks. This approach generally yields higher polysaccharide content but lower levels of triterpenoids. Specifically, polysaccharide content could be several times higher while the triterpenoid concentration in submerged *A. cinnamomea* mycelia is approximately 2.3 times lower than that observed in its fruiting body [10].

In addition to some specific metabolites, a study has demonstrated that proteins isolated from an *A. cinnamomea* strain exhibit promising anticancer activity. The FJ-01 strain was cultured on a potato-dextrose-agar (PDA) slant and incubated at 28 °C for 25 days. Subsequently, segments of the culture were transferred to a liquid seed medium (47.8 g/L corn flour, 31.9 g/L YM medium, pH 5.5) and incubated at 28 °C for an additional 7 days. A novel active protein, termed ACAP, isolated from an *A. cinnamomea* liquid fermentation medium, exhibited a dose-dependent inhibitory effect on the proliferation of HeLa and HepG2 cells. Notably, ACAP demonstrated superior efficacy compared to the positive control drug 5-Fu at low concentrations, highlighting its potential as a promising anticancer agent [11].

#### 2.1.2. Solid-State Fermentation (SSF)

Solid-state fermentation (SSF) is extensively utilized in both research and commercial production of *A. cinnamomea*, with the selection of substrates playing a pivotal role in providing essential nutrients and creating a supportive environment for fungal growth, thereby enhancing its bioactivity. Commonly used solid substrates include rice bran, wheat bran, sawdust, and agricultural residues, selected for their nutritional value and ability to simulate natural growth conditions [12]. For example, Yang et al. demonstrated that incorporating various citrus peel extracts into submerged *A. cinnamomea* cultures significantly promoted mycelial growth and polysaccharide production, and the nutrient composition of cultured mycelia closely matches that of the wild fruiting body and exhibits notable hepatoprotective potential [13]. Specifically, wheat-based solid-state fermented *A. cinnamomea* (WFAC) was prepared by inoculating liquid-cultivated *A. cinnamomea* into sterilized wheat mixed with a medium containing 2% sugar, 0.5% malt extract, and 0.5% yeast extract in water, followed by a 4-month incubation at 25 ± 2 °C. The administration of WFAC to rats demonstrated significant protective effects against CCl_4_-induced liver damage, further highlighting its therapeutic potential [13].

### 2.2. Extraction Methods for Bioactive Components of A. cinnamomea

Due to its pharmacological activities and significant therapeutic potential, *A. cinnamomea* has garnered attention for the identification and discovery of its bioactive components including polysaccharides, triterpenoids, ubiquinone derivatives, maleic and succinic acid derivatives, benzenoid derivatives, and glycoproteins. Various extraction technologies have been developed to enhance the yield of these active ingredients; these methods include liquid solvent extraction with ethanol (ETOH-E), supercritical fluid extraction (SFE) using CO_2_, high-hydrostatic-pressure extraction (HPE), ultrasonic extraction, heat reflux extraction, microwave-assisted extraction with controlled microwave power, and mechanochemical-assisted extraction (Table 1).

Water extraction tends to yield higher levels of water-soluble polysaccharides and small molecules, which are associated with immune-modulating and antioxidant activities. For instance, Tsai et al. demonstrated that the water fraction of *A. cinnamomea* possesses antioxidant properties, partly due to polysaccharides that upregulate glutathione S-transferase activity, maintain the GSH/GSSG ratio, and scavenge ROS [14]. Additionally, the water-soluble fraction from cultured mycelia of *A. cinnamomea* exhibits strong anti-inflammatory effects by inhibiting ROS production in human leukocytes at pharmacologically relevant concentrations [15].

Ethanol extraction, using varying ethanol concentrations (e.g., 50%, 70%, 95%), is effective for isolating lipophilic compounds such as polyphenols, triterpenoids, and benzoic acid derivatives. Ethanol extracts are also known for their liver-protective properties, making them suitable for medicinal and health supplement applications. For example, the ethanolic extract of *A. cinnamomea* mycelium (EMAC) has shown hepatoprotective effects through the activation of HO-1 and phase II enzymes via the MAPKs-mediated Nrf-2 signalling pathway [16]. Ethanol extracts also inhibit cancer cell migration; Chen et al. found that the ethanolic extract of *A. cinnamomea* fruiting bodies exhibits anti-migration effects in human adenocarcinoma CL1-0 cells through the MAPK and PI3K/AKT signalling pathways [17]. These highlight the value of ethanol extraction for therapeutic applications *A. cinnamomea*.

The synergistic use of advanced extraction technologies can significantly improve the efficiency and yield of *A. cinnamomea* bioactive compounds. Ultrasound-assisted extraction enhances solvent penetration, effectively extracting polysaccharides, triterpenoids, and other bioactive compounds on a large scale, while it may sometimes alter compound structures, potentially affecting bioactivity [18]. Enzyme-assisted extraction uses enzymes to break down cell walls, increasing the release of compounds like polysaccharides and proteins [19]. Despite its effectiveness, high enzyme costs limit its application to laboratory research or high-value extractions. Supercritical CO_2_ extraction, conducted at low temperatures without organic solvents, is ideal for isolating heat-sensitive, lipophilic compounds, such as triterpenoids, yielding high-purity extracts suitable for premium food and pharmaceutical products, although high equipment costs constrain large-scale implementation [20]. High-pressure-assisted extraction (HPE) offers a fast and efficient alternative to improve yield by applying high pressure to disrupt cell structures and enhance solvent penetration and operating at room temperature to preserve heat-sensitive compounds [21]. HPE achieves the desired concentration at 600 MPa in just 3 min, much faster than with ultrasound (1 h) and agitation (8 h).

**Table 1:** Using different treatment to enhance the extraction efficiency of the active components from the fruiting bodies of *A. cinnamomea*.

Entry	Type	Sample Preparation	Detection Methods	References
1	Conventional liquid solvent extraction with ethanol	Immersed in ethanol for extraction	HPLC analysis	[16,17,22]
2	Conventional shake extraction	Shake extraction at 150 rpm for 8 h	HPLC analysis	[23]
3	Supercritical CO_2_	Supercritical carbon dioxide at 35 MPa with as cosolvent	HPLC analysis	[24]
4	High hydrostatic pressure	600 MPa (100–700 MPa at 25 °C) high-pressure, liquid/solid ratio of 40:1, 3 min of treatment in a 0.3 L chamber	HPLC analysis	[21,23,25]
5	Ultrasonic extraction methods	Ultrasonic extraction at 50 Hz for 60 min	HPLC analysis	[18,23,26]
6	Heat reflux extraction	The dried powder samples were extracted with 3-fold 95% ethanol under heat reflux	UPLC	[27]
7	Mechanochemical-assisted extraction method (TAEM)	The mixture of powder and NaHCO_3_ was added into the PM-200 ball mill with steel balls (3 mm diameter, 150 g) at 300 rpm for 20 min before performing extraction by water	HPLC and LC-MS/MS	[28]

## 3. Nutritional and Physicochemical Compositions of *A. cinnamomea*

### 3.1. Nutritional Composition

The dried biomass of *A. cinnamomea* contains 6.65% moisture, 56.7% carbohydrates, 25.4% total reducing sugars, 3.92% crude ash, 9.79% crude fat, 20.1% crude fibre, and 9.49% crude protein. Also, *A. cinnamomea* is rich in sugars, amino acids, triterpenoids, polyphenols, polysaccharides, proteins, flavonoids, lignin and essential oils, enhancing its nutritional and nutraceutical value [29,30]. Supplementing a modified Potato Carrot Agar (PCA) medium with phytohormones and lignin significantly enhanced mycelial growth, increased active ingredient content, and improved antioxidant properties [31].

### 3.2. Physicochemical Profiles of A. cinnamomea

#### 3.2.1. Benzenoids

**Figure 3:**
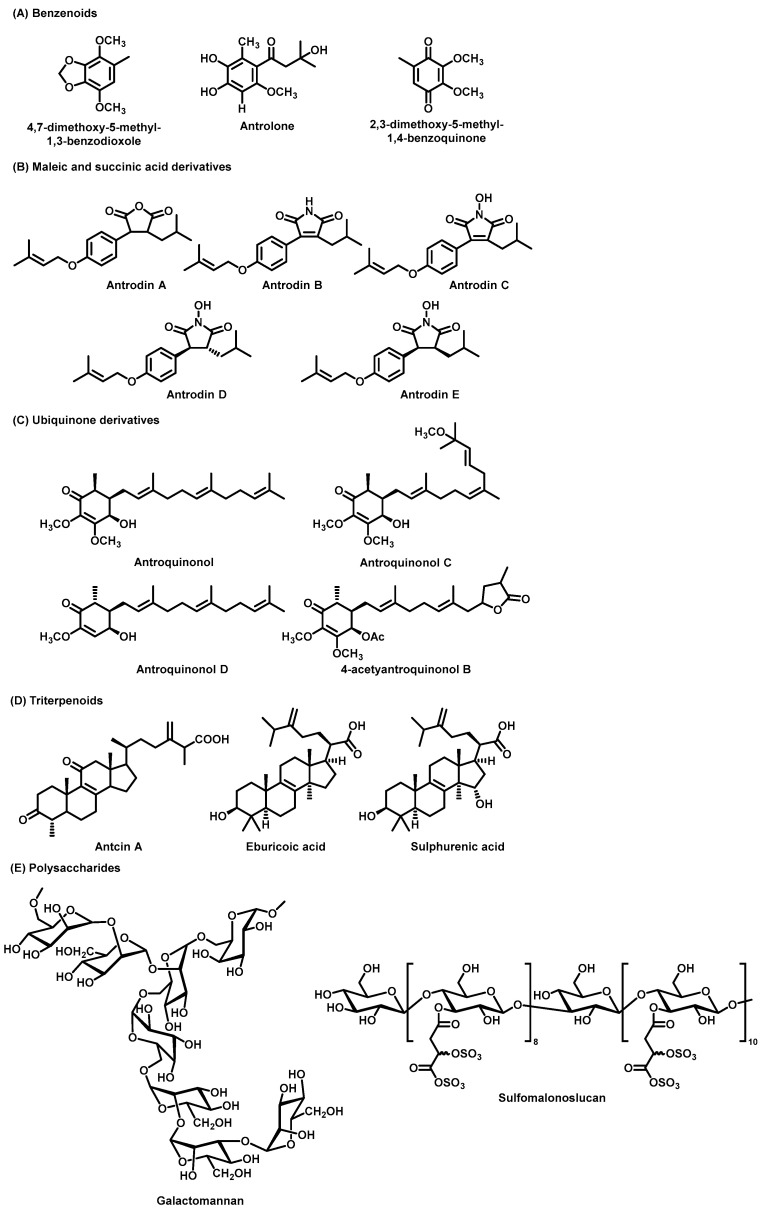
The chemical structures of the main bioactive components from *A. cinnamomea*. (**A**) Benzenoids. (**B**) Maleic and succinic acid derivatives. (**C**) Ubiquinone derivatives. (**D**) Triterpenoids. (**E**) Polysaccharides.

Several benzenoid compounds isolated from *A. cinnamomea* have been identified as possessing potent anti-inflammatory properties and have summarized key information on the preparation methods of *A. cinnamomea* components from recent studies (Figure 3). Specifically, 4,7-dimethoxy-5-methyl-1,3-benzodioxole (DMB) and antrolone have demonstrated significant anti-inflammatory effects [32,33]. Additionally, coenzyme Q0 (2,3-dimethoxy-5-methyl-1,4-benzoquinone), known for its antitumor and anti-inflammatory activities, further highlights the therapeutic potential of these compounds [34,35,36].

#### 3.2.2. Maleic and Succinic Acid Derivatives

Succinic acid, historically recognized for its use as a natural antibiotic and therapeutic agent in the 19th century, is a notable compound found in *A. cinnamomea*, which also serves as a source of maleic and succinic acid derivatives with potential hepatoprotective effects comparable to silymarin [37]. Antrodins A–E (Figure 3), maleic and succinic acid derivatives from *A. cinnamomea*, have demonstrated selective cytotoxicity against Lewis lung carcinoma (LLC) cells, highlighting their potential as candidates for cancer therapy [38]. Table 2 presents essential information on the preparation methods of maleic and succinic acid derivatives from recent studies. Furthermore, maleic acid derivatives, particularly Antrodin A, have shown protective effects against liver damage, enhancing the antioxidant and anti-inflammatory capacities of the liver while mitigating acute alcoholic liver injury [38]. Tetrodotoxin B has been identified for its anti-hepatic fibrotic effects, and Antrodin C has been found to inhibit the migration and invasion of breast cancer cells by modulating specific signalling pathways [39,40].

#### 3.2.3. Ubiquinone Derivatives

Ubiquinone derivatives are key bioactive compounds in *A. cinnamomea*, with 13 distinct derivatives isolated and characterized to date, including antroquinonol, antroquinonol B, antroquinonol C, antroquinonol D, antroquinonol L, antroquinonol M, antroquinonol Z, antrocamol LT1, antrocamol LT2, antrocamol LT3, 4-acetyantroquinonol B, 4-acetylantrocamol LT3, and antrocinnamone, have been isolated from *A. cinnamomea* [5,52]. The first ubiquinone compound identified from the solid fermentation culture of *A. cinnamomea* mycelium was antroquinonol (Figure 3) [53]. Key details on the preparation methods of ubiquinone derivatives from recent studies are summarized in Table 3. These compounds exhibit significant therapeutic potential against cancer, inflammation, periodontal disease, Parkinson’s disease, and cardiovascular disorders, supported by both in vitro models and animal models [5].

4-Acetylantroquinonol B has been evaluated for its anti-inflammatory activity, demonstrating a potent IC50 value of 14.7 μg/mL. Antroquinonol D, successfully synthesized via chemical methods, exhibits selective cytotoxicity against breast cancer cell lines (MCF7, T47D, and MDA-MB-231) with IC50 values of 8.01, 3.57, and 25.08 μM, respectively, while remaining nontoxic to normal cells. Additionally, Antroquinonol C has shown significant anticancer activity. These compounds were selected to emphasize key bioactive ubiquinone derivatives, balancing structural diversity with pharmacological relevance. Ubiquinol-10 supplementation reduces mild fatigue in healthy individuals and improves endothelium-dependent vasodilation in those with mild-to-moderate dyslipidaemia, as demonstrated by human clinical studies [54,55].

#### 3.2.4. Triterpenoids

Triterpenoids, the largest group of phytochemicals in *A. cinnamomea*, are present in significantly higher concentrations in wild strains compared to submerged-cultured counterparts [56]. Studies have identified 75 ergostanes and 28 landostanes as the predominant types in wild *A. cinnamomea*, with their levels being 10–30 times higher than those in cultivated strains [57,58], this variation underscores the need for consistent quality control methods to evaluate triterpenoid content. In *A. cinnamomea*, antcin A is recognized for its potent anti-inflammatory properties. Among the most abundant landostane-type triterpenoids, eburicoic acid, and sulphurenic acid exhibit notable bioactivities, including significant antidiabetic effects [48].

#### 3.2.5. Polysaccharides

Polysaccharides represent a highly promising class of bioactive compounds due to their diverse structural variations and functional versatility. Table 3 summarizes important information on the preparation methods of polysaccharides from recent studies. Polysaccharides isolated from *A. cinnamomea* mycelia and cultured mycelium have been extensively characterized and evaluated for their broad spectrum of biological activities. These include notable immunomodulatory and anti-inflammatory properties, as well as anticancer, cytotoxic, and anti-angiogenic effects, underscoring their therapeutic potential [59,60]. Additionally, polysaccharides have the potential to be used as natural anticancer agents as they can inhibit tumour growth by restoring or enhancing the immune system [61,62]. Polysaccharides derived from *A. cinnamomea* also demonstrate potential as natural therapeutic agents for Parkinson’s disease [63]. Chiu et al. identified a novel polysaccharide, Antrodan, isolated from the submerged fermented mycelium of *A. cinnamomea* [27]. Their study demonstrated that Antrodan, administered at a low dose of 40 mg/kg, exhibits hepatoprotective properties, potentially mediated through the activation of the AMPK/Sirt1/SREBP-1c/PPARγ signalling pathway [28]. Additionally, Antrodan has demonstrated anti-metastatic activity in the treatment of lung cancer and has been shown to mitigate cisplatin-induced renal impairment when co-administered with cisplatin [64,65]. Moreover, Antrodan exhibits promising efficacy in the treatment of non-alcoholic fatty liver disease (NAFLD), with favourable outcomes observed across various administration routes [66]. Additionally, the galactomannan ACP, characterized by an octasaccharide-repeating unit, was first isolated from this species. Perera et al. identified ACP in *A. cinnamomea* mycelia, confirming its mannose-to-galactose ratio of 75% to 25% [67].

**Table 3:** Chemical constituents and their reported activities of *A. cinnamomea*.

No.	Chemical Class	Compound Name	Cas	Formula	Biological Activity	Model	Reference
1	Diterpenes	19-Hydroxylabda8(17)-en-16,15-olide	82209-74-3	C20H32O3	–	–	[68]
2		19-Hydroxylabda8(17),13-dien-16,15-olide	82209-74-3	C20H30O3	–	–	[68]
3		14-Deoxy-11,12-didehydroandrographolide	42895-58-9	C20H28O4	Steatohepatitis and liver Injury activities	in vitro; in vivo	[68]
4		14-Deoxyandrographolide	4176-97-0	C20H30O4	Antimicrobial activity	in vitro	[68]
5		Pinusolidic acid	40433-82-7	C20H28O4	Neuroprotective and anti-inflammatory activities	in vitro	[69]
6	Terpenoids	Eburicoic acid [58]	560-66-7	C31H50O3	Hepatoprotective effects and anti-inflammatory activities	in vitro; in vivo	[70]
7		Dehydroeburicoic acid	1740-19-8	C20H28O2	Hepatoprotective, antidiabetic and antihyperlipidemic, anti-inflammatory, anti-insecticidal activities	in vitro; in vivo	[71]
8		Sulphurenic acid	–	C31H50O4	Anti-insecticidal activity	–	[54]
9		Dehydrosulphurenic acid	175615-56-2	C31H48O3	Anti-inflammatory activity	in vitro	[4]
10		15α-Acetyl-dehydrosulphurenic acid	215438	C34H50O5	Anti-inflammatory activity	in vitro	[17]
11		Versisponic acid D	–	C33H52O5	Anti-inflammatory activity	in vitro	[72]
12		3β,15α-Dihydroxylanosta-7,9(11),24-trien-21-oic acid	–	C30H46O4	Anti-inflammatory activity	in vitro	[73]
13		24-Methylenedihydrolanosterol	14297-52-2	C31H52O	–	–	[74]
14		epi-Friedelinol	16844-71-6	C30H52O	–	–	[4]
15		Antrocin	–	C15H22O2	Antioxidant, anti-mutagenic activities	in vitro	[75]
16		19-Hydroxylabda-8(17)-en-16,15-olide	–	C20H32O3	Neuroprotective activity	in vitro	[70]
17		14-Deoxy-11,12- didehydroandrographolide	42895-58-9	C20H28O4	Neuroprotective activity	in vitro	[76]
18		14-Deoxyandrographolide	4176-97-0	C20H30O4	–	–	[77]
19		Pinusolidic acid	–	C20H28O4	–	–	[78]
20		Antcin A	163597-24-8	C29H42O4	Anti-inflammatory, anti-insecticidal activities	in vitro	[52]
21		Zhankuic acid A(Antcin B)	163597-25-9	C29H40O5	Anti-inflammatory, anti-insecticidal activities	in vitro	[79]
22		Antcin C	–	C29H42O5	Anti-inflammatory activity	in vitro	[80]
23		Antcin D (Zhankuic acid F)	–	C29H40O6	–	–	[79]
24		Antcin E	–	C29H40O4	–	–	[81]
25		Antcin F	–	C29H40O5	–	–	[69]
26		Antcin G	–	C31H44O6	–	–	[69]
27		Zhankuic acid C(Antcin H)	–	C29H42O6	Anti-inflammatory, anti-insecticidal activities		[73]
28		Antcin I (Zhankuic acid B)	–	C29H42O5	Anti-inflammatory activity	in vitro	[82]
29		Antcin K	741268-13-3	C29H44O6	Anti-inflammatory activity	in vitro	[82]
30		Methyl antcinate A	169477-80-9	C30H44O4	–	–	[79]
31		Zhankuic acid B	173221-07-3	C29H42O5	–	–	[83]
32		Zhankuic acid D(Methyl antcinate B)	–	–	Anti-insecticidal activity	in vitro	[83]
33		Zhankuic acid E	–	–	–	–	[83]
34		Eburicol (24-methylenedihydrolanosterol)	6890-88-6	C31H52O	–	–	[78]
35		β-Sitosterol	83-46-5	C29H50O	–	–	[84]
36		β-Sitostenone	1058-61-3	C29H48O	–	–	[84]
37		Stigmasterol	83-48-7	C29H48O	–	–	[84]
38		Ergosta-4,6,8(14),22-tetraen-3-one	19254-69-4	C28H40O	–	–	[84]
39		Methyl antcinate	134-20-3	C8H9NO2	–	–	[85]
40		Methyl antcinate H	169477-80-9	C30H44O6	–	–	[84]
41		Eburicol	6890-88-6	C31H52O	–	–	[85]
42	Benzenoids	Antrocamphin A	–	C15H18O3	Anti-inflammatory activity	in vitro	[86]
43		Antrocamphin B	945622-08-2	C14H16O4	Anti-inflammatory activity	in vitro	[86]
44		2,3,4,5-Tetramethoxybenzoyl chloride	4521-61-3	(CH3O)3C6H2COCl	Anti-inflammatory activity	in vitro	[86]
45		Antrodioxolanone	–	C29H32O9	Anti-inflammatory activity	in vitro	[86]
46		Isobutylphenol	30749-25-8	C10H14O	–	–	[86]
47	Benzoquinone and its derivatives	5-Methylbenzodioxole-4,7-dione	7145-99-5	C8H6O4	–	–	[87]
48		2,3-Dimethoxy-5-methyl-benzoquinone	605-94-7	C9H10O4	Anti-inflammatory activity	in vitro	[87]
49		2-Methoxy-5- methyl-benzoquinone	614-13-1	C8H8O3	Antioxidant activity	in vitro	[87]
50	Maleic anhydrides	Camphorataanhydride A	656830-24-9	C19H22O4	Glycation inhibitors with lipid peroxidation activity	in vitro	[87]
51	Maleimides	Camphorataimide B	656830-25-0	C19H23NO3	Anti-breast cancer activity	in vitro	[38]
52	Maleimides	Camphorataimide C	656830-26-1	C19H23NO4	–	–	[38]
53	Lignans	Sesamin	607-80-7	C20H18O6	–	–	[38]
54		4-Hydroxysesamin	63427-86-1	C20H18O7	–	–	[40]
55	Succinic acid derivatives	Camphorataimide D	656830-26-1	C19H23NO4	–	–	[40]
56	Phenyl methanoids	4,7-Dimethoxy-5-methyl-1,3-benzodioxole	165816-66-0	C10H12O4	Anti-inflammatory and anti-tumour activities	in vitro; in vivo	[88]
57	Ubiquinone derivatives	Antroquinonol	1010081-09-0	C24H38O4	Anti-inflammatory, anti-HBV activities	in vitro	[42]
58		antroquinonol B	–	C24H36O6	Anti-inflammatory activity	in vitro	
59		antroquinonol C	–	C25H40O5	Anti-breast cancer activity	in vitro; in vivo	[16]
60		antroquinonol D	–	C23H36O3	Anti-breast cancer activity	in vitro; in vivo	[46]
61		antroquinonol L	–	C23H32O3	–	–	[53]
62		antroquinonol M	–	C23H32O3	–	–	[48]
63		antrocamol LT1	–	C24H39O5	Anti-colon cancer, anti-liver cancer, anti-kidney cancer activities	in vitro	[89]
64		antrocamol LT2	–	C26H40O6	–	–	[89]
65		antrocamol LT3	–	C24H39O5	–	–	[89]
66		4-acetyantroquinonol B	–	C26H38O7	Anti-colorectal cancer activity	in vitro	[89]
67		4-acetylantrocamol LT3	–	C26H40O6	Anticancer activity	in vitro	[89]
68		antrocinnamone	–	C23H32O3	–	–	[49]
69	Tocopherols	α-Tocospiro B	601490-41-9	C28H48O4	–	–	[89]

## 4. Pharmacological Studies on Phytochemicals Isolated from *A. cinnamomea*

To date, extensive research has elucidated the diverse bioactivities of *A. cinnamomea*. Advanced extraction techniques have facilitated the identification of various pharmacological properties, including anti-inflammatory, antitumor, and antioxidant activities [90]. These pharmacological effects will be explored in detail in the following sections, followed by a comprehensive overview of in vivo studies and human clinical trial results (Table 2 and Table 3).

### 4.1. Antrolone, 25R-Antcin A, and Versisponic Acid D: Potent Antioxidant and Anti-Inflammatory Agents for Oxidative Stress-Related Diseases

Oxidative stress, driven by reactive oxygen species (ROS), plays a key role in the pathogenesis of various diseases [91]. Antioxidant metabolites mitigate ROS-induced damage by scavenging ROS, suppressing pro-inflammatory cytokines, and modulating critical signalling pathways. Compounds such as antrolone, 25R-Antcin A, and Versisponic Acid D, isolated from *A. cinnamomea,* exhibit significant antioxidant and anti-inflammatory effects [92]. Their bioactivity underscores the therapeutic potential of *A. cinnamomea* in mitigating oxidative stress [93].

The benzenoid compound antrolone has been shown to possess significant anti-inflammatory effects by inhibiting LPS-induced production of nitric oxide (NO), prostaglandin E2 (PGE2), pro-inflammatory cytokines, and the chemokine CXCL1 in immune cells [94]. Furthermore, antrolone reduces the expression of inducible nitric oxide synthase (iNOS) and cyclooxygenase-2 (COX-2) proteins. Mechanistically, antrolone modulates the levels of Nrf2 and HO-1, which suppress the activation of NF-κB, MAPK, and AKT signalling pathways, leading to a downregulation of critical inflammatory pathways, particularly MAPK and NF-κB [95].

**Figure 4:**
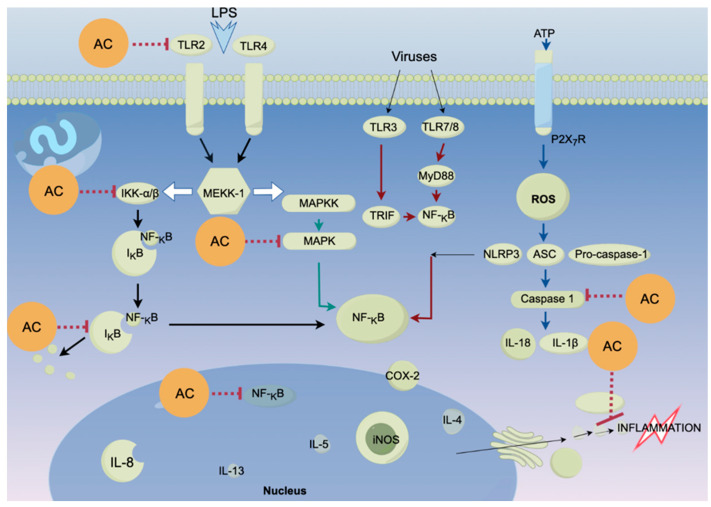
Proposed mechanisms of action of *Antrodia cinnamomea* on ROS induced oxidation. AC, *Antrodia cinnamomea* extracts. The “…I” indicates that *A. cinnamomea* exhibits anti-inflammatory bioactivity by down-regulating these proteins/molecules. Arrows mean promoting or upregulating activity. The figure was generated using Figdraw (www.figdraw.com).

Moreover, compounds such as 25R-antcin A and versisponic acid D have demonstrated efficacy in reducing LPS-induced inflammation. At a concentration of 10 μM, 25R-antcin A and versisponic acid D exhibited half-inhibitory concentrations (IC_50_) of 19.61 ± 0.8 and 17.16 ± 1.0 μM, respectively [22]. In addition to benzenoids and triterpenoids, polyphenols isolated from *A. cinnamomea* exhibit potent in vitro antioxidant activity, with maximum scavenging rates of 94.10%, 83.34%, and 95.42% against DPPH, ABTS^+^, and hydroxyl radicals, respectively, at a concentration of 0.1 mg/mL [96]. The corresponding IC_50_ values—0.01 mg/mL for DPPH, 0.014 mg/mL for ABTS^+^, and 0.007 mg/mL for hydroxyl radicals—demonstrate the remarkable antioxidant efficacy of these polyphenols [97,98]. These findings underscore the therapeutic potential of *A. cinnamomea* in relief oxidative stress and inflammation which are the underlining symptoms of all diseases (Figure 4).

### 4.2. 4-Acetylantroquinonol B, Dehydroeburicoic Acid, and Antcins: Potent Antitumor Agents for Cancer Therapy

**Figure 5:**
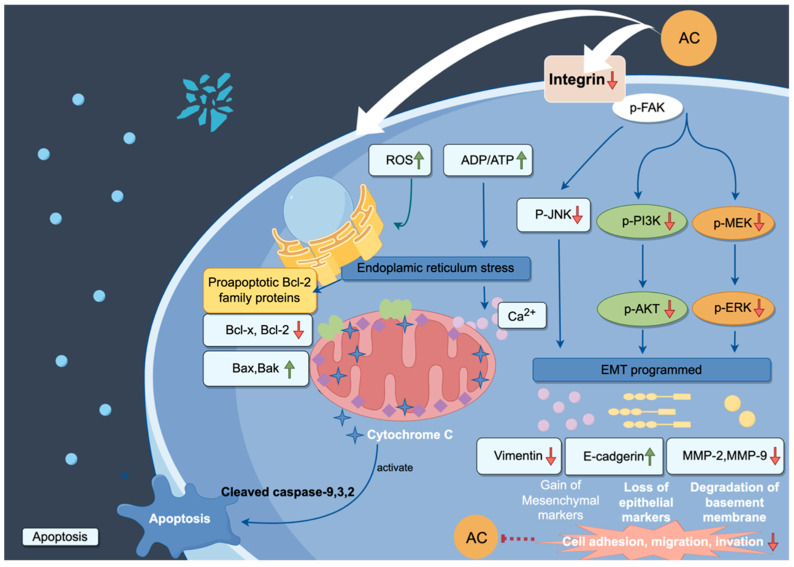
Proposed mechanisms of action of bioactive compounds from *A. cinnamomea* (AC) in the suppression of cancer. Blue arrows mean promotion. Green arrows mean up-regulation, orange arrows mean down-regulation. The figure was generated using Figdraw (www.figdraw.com).

*A. cinnamomea* extracts have diverse anticancer properties across various cell lines in vitro. For example, the ethanol extract (EEAC) inhibited the growth of A549 lung cancer cells, with an approximate IC_50_ of 170 μg/mL after 24 h of treatment, and migration via suppression of cav-1 expression and activation of p-AMPK, p21, and p27, and EEAC also suppresses ovarian carcinoma (SKOV-3) cell proliferation through HER-2/neu pathway inhibition (IC_50_ = 196 μg/mL) and demonstrates cytotoxic effects on breast cancer cells (MDA-MB-231: IC_50_ = 136 μg/mL; MCF-7: IC_50_ = 316 μg/mL) [78,99,100]. Based on the high IC_50_ values, the activity is moderate and this may be due to the presence of components that have no activity. Further purification of the active compounds would reduce the IC_50_ values. Indeed, the ubiquinone derivative 4-acetylantroquinonol B (4-AAQB) enhances dendritic cell-mediated immunity, inhibits liver cancer stem cells, and arrests HepG_2_ cell proliferation (IC_50_ = 0.1 μg/mL) by modulating p53, p21, and p27 expression, while reducing hepatocellular carcinoma metastasis [101]. Furthermore, triterpenoids and sesquiterpenoids, such as dehydroeburicoic acid (IC_50_ = 3.4–25.7 μM), antcins B, H, K, and methyl antcinates demonstrate cytotoxicity against liver (HepG2, Huh7, Hep3B), colon (HCT-116), oral (SCC-9), and breast (MCF-7, MDA-MB-231) cancer cells (IC50 = 22.3–78.0 μM) via apoptosis induction, endoplasmic reticulum stress, and the inhibition of integrin-mediated adhesion and migration [58]. These findings underscore *A. cinnamomea*’s potential as a multifunctional therapeutic agent for cancer treatment (Figure 5).

### 4.3. Antcin K and Dehydroeburicoic Acid and Eburicoic Acid as Potent Antidiabetic Agents

Diabetes mellitus stands as a significant cause of morbidity and mortality worldwide [102]. Purified triterpenoids from *A. cinnamomea* extracts, including antcin K and dehydroeburicoic acid have demonstrated potent α-glucosidase inhibitory activity, with exhibited stronger effects (EC_50_ = 0.025–0.21 mg/mL) compared to acarbose (EC_50_ = 0.278 mg/mL). These findings establish these active compounds as powerful mushroom-derived α-glucosidase inhibitors by blocking the conversion of maltose into glucose, thereby helping to regulate blood glucose levels and prevent spikes in the bloodstream [102]. Furthermore, eburicoic acid treatment increased hepatic PPARα expression, enhancing fatty acid oxidation, reducing lipid accumulation while reducing FAS expression and SREBP-1c levels, and leading to lower blood triglycerides and improved hepatic health. These studies suggest that *A. cinnamomea* mycelium powder or fruiting bodies may effectively improve insulin resistance for treating type 2 diabetes (T2D) [51].

### 4.4. Other Compounds from A. cinnamomea Extracts as Potential Therapeutic Agents for Neurological Disorders and SARS-CoV-2

Coenzyme Q0 induces ROS-mediated apoptotic and autophagic cell death, highlighting its potential effectiveness in treating central nervous system disease glioblastoma multiforme by inhibiting growth and suppressing colony formation by activating caspase-3, cleaving PARP, and dysregulating the Bax/Bcl-2 expression, also inhibits the PI3K/AKT/mTOR signalling pathway, which is crucial in apoptosis and autophagy mechanisms [79]. Furthermore, Antcin-A, Antcin-B, Antcin-C, and Antcin-I strongly show anti-acute respiratory syndrome coronavirus 2 (SARS-CoV-2) by reducing human ACE2 levels in HT-29 cells with a moderate reduction was observed in Antcin-H treated cells (5.91 ng/mL) [69].

### 4.5. In Vivo Study in Animal Models with Crude Extract

Recent research on *A. cinnamomea* extracts and pure compounds demonstrates promising anticancer effects across various cancer types and preclinical models (Table 4) [22]. *A. cinnamomea* dropping pills (ACDPs) at high (400 mg/kg/day) and low (200 mg/kg/day) doses dose-dependently inhibited tumour growth, reducing tumour weight by 48% and 67% and decreasing tumour volume without noticeable toxicity [74]. These effects were mediated by the inhibition of the PI3K/AKT signalling pathway in hepatocellular carcinoma (HCC). In leukaemia models, oral administration of an ethanol extract of *A. cinnamomea* fruiting bodies (0.9 g/kg for two weeks) reduced p-ERK1/2, p-Akt, and MMP-9 expression while increasing p21 and p27, markers of cell cycle arrest [77]. In lung cancer, ethanolic *A. cinnamomea* extracts (0.25 and 0.5 g/kg) reduced tumour size by 30–58% after 7 days and 68–76% after 14 days [78]. Compounds from *A. cinnamomea* also exhibit potent anticancer effects, 4-acetylantroquinonol B (2.5 mg/kg, intraperitoneally for 6 weeks) inhibited colorectal cancer xenograft growth by regulating the Lgr5/Wnt/β-catenin and JAK-STAT pathways, and dehydroeburicoic acid suppressed tumour growth in HL60 xenograft mice through DNA damage and mitochondrial dysfunction [103]. It also mitigated non-alcoholic fatty liver disease (NAFLD) by enhancing ALDH2 activity, promoting ROS elimination, and suppressing lipogenesis [70].

*A. cinnamomea* extracts also show therapeutic potential for nervous system disorders. CoQ0 (1.5 mg/kg, subcutaneously every two days) induced autophagy and exerted an antimetastatic effect in models of nervous system tumours [35]. In oesophageal cancer, the oral administration of *A. cinnamomea*-MFB (1.0 mg/mL for 34 days) enhanced the effectiveness of radiation therapy, delaying tumour growth [104]. These findings demonstrate *A. cinnamomea* potential as a multi-targeted therapeutic agent, with anticancer and neuroprotective effects mediated through mechanisms such as PI3K/AKT inhibition, cell cycle regulation, autophagy induction, and mitochondrial dysfunction.

### 4.6. Human Clinical Trials on Antrodia cinnamomea Extracts

Some clinical trials have highlighted the therapeutic potential of *A. cinnamomea* across multiple health domains (Table 5). *A. cinnamomea* extracts and metabolites exhibit significant immunomodulatory, hepatoprotective, antihypertensive, and cholesterol-lowering effects, supporting its broad medicinal applications. A Phase I study of LEAC-102, an *A. cinnamomea*-derived extract, assessed its safety and immunomodulatory effects in 18 healthy adults (20–44 years) with escalating doses up to 2988 mg/day [105]. LEAC-102 showed a favourable safety profile with no serious adverse effects and enhanced T-cell activation, upregulating PD-1 expression, suggesting potential utility in cancer immunotherapy.

Daily supplementation with *A. cinnamomea* mycelium resulted in significant reductions in systolic and diastolic blood pressure, attributed to plasma renin activity inhibition and reduced oxidative stress, indicating its potential as an antihypertensive agent. Another study of 28 participants with non-alcoholic steatohepatitis (NASH) showed that 420 mg *A. cinnamomea* mycelium daily for 3–6 months significantly reduced liver steatosis and TNF-α levels without adverse events, demonstrating its hepatoprotective efficacy [106,107]. For cholesterol management, 36 participants consuming 380 mg of AC mycelium (LAC) capsules twice daily for 3 months showed modest reductions in total cholesterol without adverse effects on liver or renal function [108]. Additionally, 44 Japanese adults taking 250 mg *A. cinnamomea* mycelium extract daily for 12 weeks experienced improved liver health, particularly in individuals with regular alcohol consumption [109].

These studies underscore the safety and efficacy of *A. cinnamomea* as a therapeutic agent with broad applications in immune modulation, liver protection, blood pressure regulation, and cholesterol management, providing a strong foundation for further clinical research.

## 5. Industrial Applications and Quality Control Issues of *A. cinnamomea* Extract Products

The development of natural products with verified bioactivity, such as *A. cinnamomea*, offers significant potential for advancing public health. Industrial applications of *A. cinnamomea* span a wide range of functional foods and nutraceuticals, including wines (CN104017703A), tablets (CN103784482B), dripping pills (WO2016184147A1), tea (TW201221065A), and soft confectioneries (CN104186880A) enriched with its bioactive constituents. Furthermore, *A. cinnamomea* extracts have demonstrated potential therapeutic effects, including the inhibition of cariogenic and periodontopathic bacteria such as *Streptococcus mutans* and *Porphyromonas gingivalis*, thereby mitigating dental plaque formation and offering oral health benefits(CN104127355A).

Despite its broad applications, the commercialization of *A. cinnamomea* products is hindered by significant quality control challenges. The content of bioactive triterpenoids, a key indicator of product efficacy, varies substantially between wild and cultivated strains. Additionally, many commercial products are derived from lower-quality mycelium rather than fruiting bodies, resulting in diminished bioactive compound profiles. Current quality control practices, often limited to assessing a narrow range of marker compounds (e.g., antcin A, B, C, K) using HPLC, fail to address broader variations and may lack sensitivity. Issues such as insufficient standardization exacerbate the inconsistency and unreliability of *A. cinnamomea* products. These shortcomings highlight the urgent need for comprehensive and robust quality control methodologies to ensure consistency, authenticity, and efficacy across *A. cinnamomea*-based products. To ensure the quality and consistency of *A. cinnamomea*-derived products on the market, always recommend including key triterpenoids as marker compounds. Due to the significant presence of terpenoids, which account for 63% of the composition in the fruiting bodies of *A. cinnamomea*, this group of natural compounds has become the subject of numerous phytochemical studies. The identified triterpenoids—antcin K, (25S)-antcin H, (25R)-antcin H, (25R)-antcin C, (25S)-antcin C, (25R)-antcin A, 15α-acetyl-dehydrosulphurenic acid, versisponic acid D, dehydroeburicoic acid, and eburicoic acid—serve as essential indicators of bioactive content in *A. cinnamomea*. These compounds were selected for their significant pharmacological activities, particularly their promising anti-inflammatory effects, and can serve as reliable indicators of bioactive content in commercial formulations. To ensure reliable quantification, also had study developed validated HPLC-UV method demonstrating high sensitivity, linearity, precision, and accuracy [110]. Incorporating these triterpenoids into the quality control framework can help mitigate the variability often observed in the market and enhance the standardization and reliability of *A. cinnamomea* products. We hope this method offers a rigorous solution to the quality control challenges associated with the highly valued *A. cinnamomea* food and nutraceutical products, which often exhibit significant variability and inconsistency, thereby contributing to the standardization and reliability of these products in the market.

## 6. Summary and Future Perspectives

Pharmacologically active natural compounds have long been recognized for their roles in drug discovery and development. *A. cinnamomea*, an emerging medicinal fungus, is known for its diverse biological activities and therapeutic potential. Despite significant progress in isolating bioactive compounds from its fruiting bodies and cultured mycelia, several challenges persist, hindering its full application in nutraceuticals and pharmaceuticals.

A primary limitation lies in the inefficiency of conventional solvent extraction methods, which are often characterized by high solvent consumption, low yields, and adverse environmental impacts. The health risks associated with organic solvents further emphasize the need for sustainable and efficient extraction technologies. Future studies should focus on developing environmentally friendly solvents and optimizing extraction processes to enhance yield while minimizing ecological and health concerns. The systematic identification of characteristic metabolites in *A. cinnamomea* remains inadequate, with current techniques for isolation, purification, and characterization limiting the development of high-quality products. Establishing advanced and standardized methodologies for metabolite profiling is critical to facilitate the industrial application of *A. cinnamomea* as a reliable source of bioactive compounds. Finally, the mechanisms underlying the pharmacological activities of specific *A. cinnamomea* compounds remain poorly understood. The research to date has largely relied on crude extracts or compound mixtures, complicating the identification of individual roles. Understanding the synergistic, antagonistic, or independent effects of these bioactive compounds is essential to ensure efficacy and safety. The isolation and characterization of pure compounds will enable the elucidation of their mechanisms of action, facilitating their development as targeted therapeutic agents.

Addressing these challenges through innovative approaches in extraction technologies, metabolite profiling, and mechanism-based studies will unlock the full potential of *A. cinnamomea* as a nutraceutical and pharmaceutical resource. Furthermore, integrating sustainable practices, such as waste valorization, will enhance its environmental and economic viability. These advancements will not only support the evidence-based application of *A. cinnamomea* but also contribute to the development of next-generation nutraceuticals and therapeutics for global health challenges.

## Figures and Tables

**Table 2:** Preparation methods of *A. cinnamomea* components in recent studies.

	Sources	Extraction Method	Isolation and Purification Method	References
Benzenoids				
4,7-Dimethoxy-5-methyl-1,3-benzodioxole	Mycelia	Extracted with methanol for 24 h at room temperature	Silica gel column and Sephadex LH-20 column	[41]
Antrolone	Mycelia	Extracted with 95% ethanol for 24 h at room temperature	Silica gel column chromatography and medium-pressure liquid chromatography	[33]
2,3-Dimethoxy-5methyl-1,4-benzoquinone	Mycelia	Extracted with 95% ethanol for 24 h at room temperature	Semipreparative HPLC	[35]
Maleic and succinic acid derivatives				
Antrodin A	Mycelia	Extracted with absolute ethanol at a ratio of 1:100 (g/mL), the ethanol extract was then extracted twice with ethyl acetate/water (1:1)	Silica gel column chromatography in a Reveleris PREP purification system	[42]
Antrocin B	Mycelia	Extracted with methanol for 24 h at room temperature	Silica gel column and semipreparative HPLC	[39]
Antrocin C	Mycelia	Extracted in methanol and then partitioned with n-hexane chloroform and ethyl acetate	Silica gel column and semipreparative HPLC	[43]
Antrocin D	Mycelia	Extracted with methanol for 24 h at room temperature	Silica gel column and semipreparative HPLC	[43]
Antrocin E	Mycelia	Extracted with methanol for 24 h at room temperature	Silica gel column and semipreparative HPLC	[43]
Ubiquinone derivatives				
Antroquinonol	Mycelia (solid-state); Fruiting bodies	Extracted three time with n-hexane by stirring at room temperature for 6 h	Silica-gel gravity column (230–400 mesh, 5 × 45 cm^2^) and Sephadex LH-20 (5 × 70 cm^2^)	[44]
Antroquinonol C	Mycelia	Extracted with 95% alcohol for 24 h at room temperature	Semipreparative HPLC	[45]
Antroquinonol D	Mycelia	Extracted with 95% alcohol for 24 h at room temperature	Semipreparative HPLC	[46]
4-acetyantroquinonol B	Mycelia	Extracted with ethyl acetate	HPLC and silica gel column (4.6 × 250 mm^2^)	[47]
Triterpenoids				
Antcin A	Fruiting bodies	Extracted with MeOH at room temperature for 7 days	Silica gel column and semi-preparative HPLC	[48]
Eburicoic acid	Fruiting bodies	Extracted with methanol at room temperature for 4 days and then partitioned (three times) with ethyl acetate	Silica gel and HPLC	[49]
Sulphurenic acid	Fruiting bodies	Extracted three times with methanol at room temperature (4 days × 3)	Silica gel and HPLC	[50]
Polysaccharides				
Galactomannan	Mycelia	Extracted with cold water and lyophilized to obtain crude polysaccharides (recovery percentage was 8.29 *w*/*w*%)	Mycelia of A. cinnamomea were fermented, lyophilized, and then ground into powder	[51]
Galactose	From Galactomannan	Lyophilizing purified ACP Purification of *A. cinnamomea* Polysaccharides	Gel filtration chromatography HW65F column	[51]
Mannose	From Galactomannan	Lyophilizing purified crude polysaccharide	Gel filtration using chromatography HW65F column	[51]

**Table 4:** In vivo bioactivity of *A. cinnamomea* extracts.

Type	Subjects	Oral Administration Dosage	Key Findings	Refs.
Hepatocellular carcinoma (HCC)	Eight-week-old BALB/c mice	ACDPs at high (400 mg/kg/day) and low (200 mg/kg/day) doses	ACDPs dose-dependently inhibited tumour growth and significantly decreased tumour volume	[74]
Leukaemia	BALB/c mice allograft tumour model	Ethanol extract of *A. cinnamomea* fruiting bodies (0.9 g kg^−1^, orally administered for 2 weeks	Reducing p-ERK1/2, p-Akt and MMP-9, and upregulating p21 and p27	[77]
Lung cancer	C57BL/6J allograft tumour model	Ethanolic extracts from *Antrodia cinnamomea* (0.25 and 0.5 g/kg).	Treatment of EEAC markedly reduced tumour size	[78]
Nervous system	Female 5–6 weeks old BALB/c-nu	Rodent chow and water	CoQ0 was effective in the reduction in tumour formation	[81]
Nervous system	Female BALB/c-nu mice	CoQ0 (1.5 mg/kg, administered subcutaneously every 2 days)	CoQ0 treatment induced autophagy and autophagy-mediated antimetastatic effects	[77]
Esophageal cancer cells	Specific pathogen-free male BALB/c nude mice (4 weeks old, 25–28 g)	AC-MFB or with 100 μL of a normal saline.	Have a synergistic effect on the tumour growth delay	[104]

**Table 5:** Human trials bioactivity of *A. cinnamomea* extracts.

Participants	Study Design	Key Findings	Refs.
Eighteen healthy participants.	Volunteers were divided into five cohorts (dose levels A, B, C, D, E), with up to six evaluable healthy volunteers per cohort, to assess the maximum tolerated dose (MTD) of LEAC-102.	LEAC-102 showed potential immunomodulatory effects by promoting adaptive T-cell activation and dose-dependently upregulating PD-1 expression.	[105]
28 Participants.	Twenty-eight participants were treated with three capsules per day containing either 420 mg of ACM or 420 mg of starch as a placebo.	The ACM group showed reduced steatosis and TNF-α levels after three and six months, indicating a hepatoprotective effect in NASH, with no adverse events reported.	[106]
A random allocation sequence for assigning participants.	Participants were treated with capsules per day containing either 420 mg of *A. cinnamomea* mycelium.	Eight weeks of AC mycelium treatment reduced SBP, DBP, and oxidative stress by inhibiting PRA, suggesting it as a safe alternative for mildly hypertensive, unmedicated patients.	[107]
36 Eligible participants.	Participants took two LAC capsules (380 mg each of A. cinnamomea solid-state mycelium) twice daily for three months.	LAC reduced marginally high cholesterol without adverse effects on liver or kidney function.	[108]
Forty-four eligible Japanese adults.	Taking an ACME capsule (250 mg of ACME powder) or a placebo capsule.	ACME might effectively improve liver health in regular drinkers.	[109]

## Data Availability

No new data were created or analyzed in this study. Data sharing is not applicable to this article.
